# Virtual acoustics in inhomogeneous media with single-sided access

**DOI:** 10.1038/s41598-018-20924-x

**Published:** 2018-02-06

**Authors:** Kees Wapenaar, Joeri Brackenhoff, Jan Thorbecke, Joost van der Neut, Evert Slob, Eric Verschuur

**Affiliations:** 1 Department of Geoscience and Engineering, Delft University of Technology, Stevinweg 1, 2628 CN Delft, The Netherlands; 2 Department of Imaging Physics, Delft University of Technology, Lorentzweg 1, 2628 CJ Delft, The Netherlands

## Abstract

A virtual acoustic source inside a medium can be created by emitting a time-reversed point-source response from the enclosing boundary into the medium. However, in many practical situations the medium can be accessed from one side only. In those cases the time-reversal approach is not exact. Here, we demonstrate the experimental design and use of complex focusing functions to create virtual acoustic sources and virtual receivers inside an inhomogeneous medium with single-sided access. The retrieved virtual acoustic responses between those sources and receivers mimic the complex propagation and multiple scattering paths of waves that would be ignited by physical sources and recorded by physical receivers inside the medium. The possibility to predict complex virtual acoustic responses between any two points inside an inhomogeneous medium, without needing a detailed model of the medium, has large potential for holographic imaging and monitoring of objects with single-sided access, ranging from photoacoustic medical imaging to the monitoring of induced-earthquake waves all the way from the source to the earth’s surface.

## Introduction

In many acoustic applications, ranging from ultrasonics to seismology, virtual sources can be created by emitting a focusing wave field from the boundary into the medium^1–4^. Time-reversal mirroring, developed by Fink and co-workers^3,4^, is a well-known approach to create a virtual source. It exploits the fact that the wave equation in a lossless medium is symmetric in time. In many practical situations, like in non-destructive testing^5–9^, medical imaging^10,11^, near-field acoustic holography^12–14^ or geophysical holography^15–17^, the medium can be accessed from one side only. In those cases the time-reversal approach is not exact, and it breaks down in inhomogeneous media with strong impedance contrasts. Recent work by the authors^18–20^ and others^21–24^ concerns the design of single-sided focusing functions. When emitted from the upper boundary into the medium, these focusing functions yield well-defined foci at predefined positions, which act as omnidirectional virtual sources. This work is inspired by the Marchenko equation of quantum mechanics^25–27^ and its applications in 1D autofocusing^28–30^.

We start this paper with a comparison of the time-reversal method and the single-sided focusing approach, at the hand of a number of numerical examples. Next, we discuss our approach for retrieving virtual sources and receivers from single-sided reflection data. We apply this methodology to ultrasonic physical model data and seismic reflection data. Finally, we discuss potential applications for photoacoustic medical imaging and for monitoring of induced-earthquake waves.

## Time-reversal versus single-sided focusing

**Figure 1:**
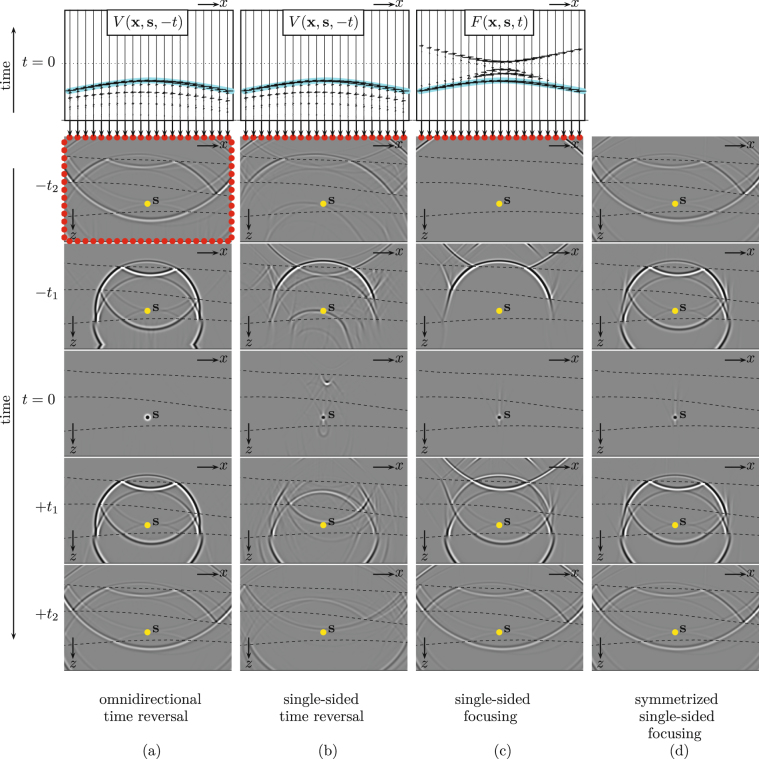
Illustration of virtual-source methods. (**a**) A time-reversed point source response is emitted from the enclosing boundary into the inhomogeneous medium. For negative time, it converges towards the focal point, where it focuses at *t* = 0. Subsequently, the focal point acts as an omnidirectional radiating virtual source. (**b**) Emission of the time-reversed response from the upper boundary only. Ghost foci occur at *t* = 0. The virtual source radiates mainly downward. (**c**) Emission of a single-sided focusing function from the upper boundary only. No ghost foci occur at *t* = 0. The virtual source radiates mainly downward. (**d**) Symmetrizing the previous result. No ghost foci occur at *t* = 0. The virtual source is omnidirectional.

The time-reversal method is illustrated in the first column of Fig. 1, for a lossless layered medium with curved interfaces (denoted by the dashed lines in the grey panels) and different propagation velocities and mass densities in the layers between these interfaces. The top panel shows the time-reversal of the response *V*(**x**, **s**, *t*) to a point source at **s** in the third layer of the medium, as a function of receiver position **x** = (*x*, *z*) along the boundary and time *t*. *V* stands for the normal component of the particle velocity. Only the response at the upper boundary is shown, but the response is available along the entire enclosing boundary ${\mathbb{S}}$. The time-reversed response *V*(**x**, **s**, −*t*) is fed to sources (the red dots) at the original positions of the receivers, which emit the wave field back into the medium. The other panels in column (a) show “snapshots” (i.e., wave fields frozen at constant time) of the wave field propagating through the medium. For negative time (… −*t*_2_, −*t*_1_ …), the field follows the same paths as the original field, but in opposite direction. Then, at *t* = 0, the field focuses at the position **s** of the original source. Because there is no sink to absorb the focused field, the wave field continues its propagation, away from the focal point. Hence, the focal point acts as a virtual source. The snapshots for positive time (… +*t*_1_, +*t*_2_ …) show the response to this virtual source. The virtual source is omni-directional and radiates a perfect replica of the original field into the inhomogeneous medium. Mathematically, time-reversal acoustics is formulated as follows^31^:

1 $$G({\bf{r}},{\bf{s}},t)+G({\bf{r}},{\bf{s}},-t\mathrm{)=2}{\oint }_{{\mathbb{S}}}\mathop{\underbrace{G({\bf{r}},{\bf{x}},t)}}\limits_{ \textquotedbl propagator \textquotedbl }\ast \mathop{\underbrace{V({\bf{x}},{\bf{s}},-t)}}\limits_{ \textquotedbl secondary\,sources \textquotedbl }\,{\rm{d}}{\bf{x}}$$

(see Supplementary Information). On the right-hand side, the time-reversed field *V*(**x**, **s**, −*t*) is propagated through the medium by the Green’s function *G*(**r**, **x**, *t*) from the sources at **x** on the boundary ${\mathbb{S}}$ to any receiver position **r** inside the medium (the asterisk denotes convolution). The integral is taken along all sources **x** on the closed boundary. Note that the right-hand side resembles Huygens’ principle, which states that each point of an incident wave field acts as a secondary source, except that here the secondary sources on ${\mathbb{S}}$ consist of time-reversed measurements rather than an actual incident field. On the left-hand side, the time-reversed Green’s function *G*(**r**, **s**, −*t*) represents the wave field at negative time that converges to the focal point **s**; the Green’s function *G*(**r**, **s**, *t*) is the response at positive time to the virtual source at **s**.

Figure 1(b) shows what happens when the time-reversed response is emitted into the medium by sources (red dots) at the upper boundary only. The field still focuses at *t* = 0, but in addition several ghost foci occur at *t* = 0. The field at positive time is a virtual-source response, contaminated by artefacts, caused by the ghost foci. Moreover, because the focal point is illuminated mainly from above, the virtual source is far from isotropic: it radiates mainly downward.

We now introduce the single-sided focusing approach, which is designed to overcome the limitations of the time-reversal approach in inhomogeneous media with strong impedance contrasts. The upper panel in Fig. 1(c) shows a 2D focusing function *F*(**x**, **s**, *t*), for the same focal point **s** as in the time-reversal example. Note that the main event (indicated in blue) is the same as that in *V*(**x**, **s**, −*t*) in the upper panel in Fig. 1(b), but the other events in *F*(**x**, **s**, *t*) come after the main event (instead of preceding it, like in *V*(**x**, **s**, −*t*)). The snapshots in Fig. 1(c) show the propagation of this focusing function through the medium. Mathematically, the emission of the focusing function *F*(**x**, **s**, *t*) into the medium by sources at **x** at the upper boundary ${{\mathbb{S}}}_{0}$ is described by

2 $$G({\bf{r}},{\bf{s}},t)+\,{\rm{a}}{\rm{n}}{\rm{t}}{\rm{i}} \mbox{-} {\rm{s}}{\rm{y}}{\rm{m}}{\rm{m}}{\rm{e}}{\rm{t}}{\rm{r}}{\rm{i}}{\rm{c}}\,{\rm{a}}{\rm{r}}{\rm{t}}{\rm{e}}{\rm{f}}{\rm{a}}{\rm{c}}{\rm{t}}{\rm{s}}\,={\int }_{{{\mathbb{S}}}_{0}}G({\bf{r}},{\bf{x}},t)\ast F({\bf{x}},{\bf{s}},t){\rm{d}}{\bf{x}}$$

(see Supplementary Information). The right-hand side resembles again Huygens’ principle, this time with the focusing function defining secondary sources on ${\mathbb{S}}$_0_ only. The left-hand side represents the virtual-source response *G*(**r**, **s**, *t*), contaminated by artefacts that are anti-symmetric in time. Because the anti-symmetric term vanishes a*t t* = 0, the panel a*t t* = 0 in Fig. 1(c) shows a “clean” focus. Like in the time-reversal method, the focused field acts as a virtual source. The snapshots at positive time show that this virtual source radiates mainly downward.

Next, we symmetrize both sides of equation (2), by adding the time-reversal. This suppresses the anti-symmetric artefacts:

3 $$G({\bf{r}},{\bf{s}},t)+G({\bf{r}},{\bf{s}},-t)={\rm{Symmetrize}}({\int }_{{{\mathbb{S}}}_{0}}G({\bf{r}},{\bf{x}},t)\ast F({\bf{x}},{\bf{s}},t){\rm{d}}{\bf{x}})$$

(see Supplementary Information). Note that the left-hand side is identical to that in equation (1). However, unlike equation (1), the right-hand side of equation (3) contains an integral along the accessible boundary ${\mathbb{S}}$_0_ only. Symmetrization implies addition of the snapshots at negative times in Fig. 1(c) to those at the corresponding positive times and vice versa, see Fig. 1(d). Note that these superposed snapshots are nearly identical to those obtained by emitting the time-reversed response into the medium from the entire enclosing boundary (Fig. 1(a)). The remaining artefacts are caused by the finite source aperture and the fact that evanescent waves are neglected in equations (2) and (3) (see Supplementary Information).

## Retrieving virtual sources and receivers from single-sided reflection data

### Virtual acoustics methodology

The snapshots in Fig. 1 (for both methods) were obtained by numerically modelling the medium’s response to fields emitted from (parts of) its boundary. These snapshots nicely visualise the propagation, scattering, focusing and defocusing of the fields inside the medium. In practical situations these fields are not visible, unless receivers would be placed throughout the medium, which is of course not feasible. However, our focusing methodology can be extended to create not only virtual sources, but also virtual receivers anywhere inside the medium. As input we need the reflection response of the medium, measured with sources and receivers at the accessible boundary ${\mathbb{S}}$_0_ only (hence, no physical sources nor receivers are needed inside the medium). The reflection response is represented by the Green’s function *G*(**x**′, **x**, *t*), where **x** denotes the variable position of the source and **x**' that of the receiver, both at ${{\mathbb{S}}}_{0}$. Consider the following variant of equation (3)

4 $$G({\bf{r}},{\bf{x}},t)+G({\bf{r}},{\bf{x}},-t)={\rm{Symmetrize}}({\int }_{{{\mathbb{S}}}_{0}}G({\bf{x}}\text{'},{\bf{x}},t)\ast F({\bf{x}}^{\prime} ,{\bf{r}},t){\rm{d}}{\bf{x}}^{\prime} )$$

(see Supplementary Information). This expression shows how the recorded data *G*(**x**′, **x**, *t*), measured at the upper boundary of the medium, are transformed into *G*(**r**, **x**, *t*) and its time-reversal, being the response to a real source at **x**, observed by a virtual receiver at **r** anywhere inside the medium. The focusing function *F*(**x**′, **r**, *t*), required for this transformation, can be derived from the recorded data *G*(**x**′, **x**, *t*), using the multidimensional Marchenko method^18–20,32,33^. We have implemented a 2D version of the Marchenko method as an iterative process^34^. The time-reversal of the direct arriving wave between **x**′ and **r** is used as an initial estimate of the focusing function *F*(**x**′, **r**, *t*). This direct arrival, in turn, is based on an estimate of the propagation velocity of the medium. This does not require information about the layer interfaces, nor about the internal structure of the layers: a smooth background model suffices to compute the direct arrival^21^. Note that estimating a background model is state-of-the-art methodology in geophysical imaging^35^. Then, by evaluating equation (4) we obtain *G*(**r**, **x**, *t*) for any virtual receiver position **r** inside the medium. Next, using the retrieved virtual-receiver data *G*(**r**, **x**, *t*) in the right-hand side of equation (3), we obtain *G*(**r**, **s**, *t*) and its time-reversal, being the response to a virtual source at **s**, observed by virtual receivers at **r**.

Theoretical research shows that this methodology can be generalised for vectorial wave fields in lossless media, such as electromagnetic waves, elastodynamic waves (after decomposition at the surface into *P*- and *S*-waves), etc^36,37^. Small to moderate propagation losses can be accommodated by applying loss corrections to the data before applying the Marchenko method^38^.

In the following we apply the virtual acoustics methodology for scalar wave fields in lossless media, as outlined above, to ultrasonic physical model data and seismic reflection data.

### Application to ultrasonic physical model data

**Figure 2:**
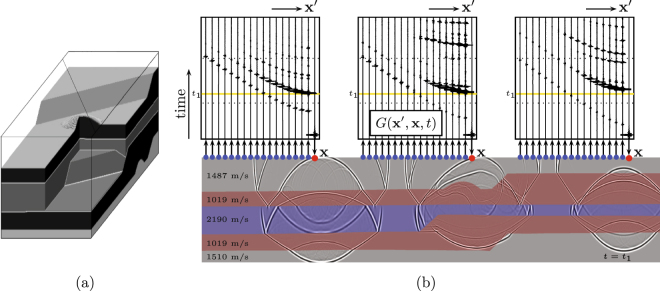
(**a**) 3D physical model. The grey-levels indicate different propagation velocities and mass densities. Ultrasonic reflection experiments are carried out along the diagonal line above the model. (**b**) 2D cross-section of the physical model (with modelled snapshots, for visualisation only) and the actually recorded response at the surface, *G*(**x**′, **x**, *t*) (here shown for 3 source positions **x** and 3 × 17 receiver positions **x**′).

Figure 2(a) shows a 3D physical model, composed of silicone gel and beeswax layers with different acoustic propagation velocities (their numerical values are tabulated in Fig. 2(b)). The size of the model is 70 × 600 × 600 mm. The model is placed in a watertank and probed with ultrasound, emitted and received by piezo-electric transducers in the water. The acquisition is carried out along a horizontal diagonal line (indicated in Fig. 2(a)), 12 mm above the upper boundary of the model and perpendicular to its main structures. A 2D cross-section of the model below the acquisition line is shown in Fig. 2(b). The emitting transducer sends a sweep signal in the frequency range 0.4 MHz to 1.8 MHz. The resulting wave field propagates through the water into the model, propagates and scatters inside the model, and propagates back through the water to the acquisition line, where it is recorded by a receiving transducer. The recorded response is deconvolved for the sweep signal, effectively compressing the source signal to a short zero-phase pulse with a central frequency of 1.1 MHz^39^. This experiment is repeated 106 times, with the source at the same position and the receiver moving along the acquisition line in steps of 1.25 mm. Next, the source is moved 1.25 mm along the line and again 106 traces are recorded. This whole process is carried out 301 times, leading to a recorded reflection response consisting of 301 × 106 = 31 906 traces. Figure 2(b) shows 51 of those traces, for 3 source positions and 17 receivers per source position. Before further processing, source-receiver reciprocity is applied, effectively doubling the number of traces, and the data are interpolated to a twice as dense spatial grid (source and receiver spacing 0.625 mm) to suppress spatial aliasing.

**Figure 3:**
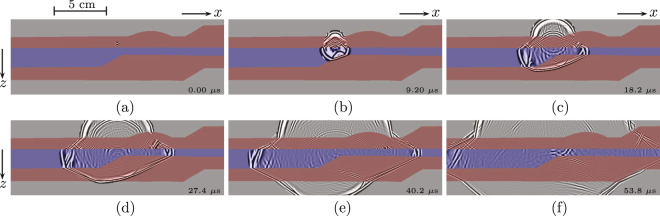
Virtual response *G*(**r**, **s**, *t*) + *G*(**r**, **s**,−*t*), retrieved from the single-sided ultrasonic reflection response *G*(**x**′, **x**, *t*) of the physical model in Fig. 2(a). (**a**) *t* = 0 *μ*s. (**b**) *t* = 9.2 *μ*s. (**c**) *t* = 18.2 *μ*s. (**d**) *t* = 27.4 *μ*s. (**e**) *t* = 40.2 *μ*s. (**f**) *t* = 53.8 *μ*s.

We denote the recorded reflection response by Green’s function *G*(**x**′, **x**, *t*), where **x** denotes the variable position of the source and **x**′ that of the receiver (actually the recorded response is the Green’s function convolved with the compressed source pulse, but for the sake of simplicity we treat the recorded data as a Green’s function). We apply the methodology discussed above to this response. Figure 3 shows snapshots of the virtual acoustic response *G*(**r**, **s**, *t*) + *G*(**r**, **s**, −*t*), for a fixed virtual source inside the second layer of the 3D physical model and variable virtual receiver positions **r** throughout the 2D cross-section of the model. The different colours in the background of this figure indicate the different layers. We used the velocities of these layers to model the direct arrivals, as initial estimates for the focusing functions. Note, however, that we did not use information about the layer interfaces for the retrieval of the virtual response: all scattering information comes directly from the recorded reflection response. The figure clearly shows the evolution of the wave field through the medium, including scattering at the layer interfaces. Imperfections are explained by the finite aperture, the limited radiation angles of the piezo-electric transducers, the negligence of evanescent waves and the fact that we used a 2D method to retrieve this virtual wave field in a 3D medium.

### Application to seismic reflection data

**Figure 4:**
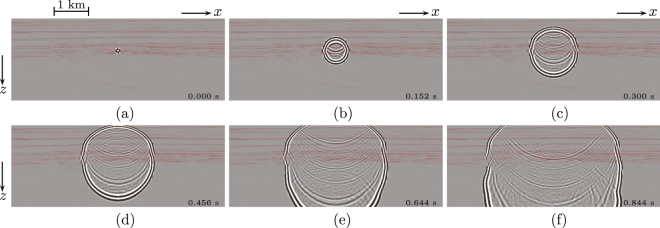
Virtual response *G*(**r**, **s**, *t*) + *G*(**r**, **s**,−*t*), retrieved from the single-sided seismic reflection response *G*(**x**′, **x**, *t*) of the Vøring Basin. (**a**) *t* = 0 ms. (**b**) *t* = 152 ms. (**c**) *t* = 300 ms. (**d**) *t* = 456 ms. (**e**) *t* = 644 ms. (**f**) *t* = 844 ms.

The proposed methodology can be applied to reflection data at a wide range of scales. Next we apply our methodology to vintage seismic reflection data, acquired in 1994 over the Vøring Basin by SAGA Petroleum A.S. (currently part of Statoil ASA). We use a smooth background model to define the initial estimates of the focusing functions. Figure 4 shows snapshots of *G*(**r**, **s**, *t*) + *G*(**r**, **s**, −*t*) obtained from these seismic data. Again, the evolution of the retrieved wave field clearly includes the primary and multiply scattered events, which have been obtained directly from the recorded reflection data. In the background these snapshots show an independently obtained seismic image of the interfaces between the geological layers, for visualisation only. Note the consistency between the position of these interfaces and the apparent origin of scattering in the snapshots.

## Discussion

The ability to create virtual sources and receivers inside a medium from single-sided reflection data opens new ways for imaging and monitoring. An exciting new field in medical imaging is photoacoustic (PA) imaging^40^, a method which employs the conversion of optical energy into acoustic energy at those locations inside the medium where light is absorbed. The resulting acoustic wave field may be very complex because usually many PA sources go off simultaneously and inhomogeneities in the medium may cause reflection artefacts^41^. Our proposed virtual acoustics methodology could be applied to ultrasonic reflection measurements to predict the direct and scattered wave fields of (clusters of) virtual PA sources, thus improving the interpretation and imaging of the complex wave field of actual PA sources. With the emergence of dual-modality ultrasound and photoacoustic imaging tools^42^ this becomes feasible and the first steps in this direction have already been made^33^. Note that in medical applications it is often sufficient to use a homogeneous background model, which means that analytical expressions can be used for the initial estimate of the focusing functions. Real-time application of our virtual acoustics methodology for medical imaging therefore seems feasible, particularly when the imaging is restricted to a finite region of interest.

Another exciting potential application is the investigation of induced seismicity. By acquiring high-resolution seismic reflection data in areas prone to induced seismicity, our virtual acoustics approach could forecast the wave field and the associated ground motion caused by possible future earthquakes. Moreover, when the same acquisition system is also used to passively record the response to actual induced earthquakes, our method could be used to create virtual seismometers in the subsurface around the actual earthquake and use these to retrieve accurate knowledge of the source mechanism of the earthquake, insight in the evolution of the geomechanical state of the subsurface (horizontal and vertical stress distribution, fault and fracture properties etc.), and deep understanding of the link between the earthquake and the observed ground motion.

### Data availability

The source code that was used to generate Fig. 1, including the Marchenko method, can be downloaded from https://github.com/JanThorbecke/OpenSource. The physical model dataset analysed in Figs 2 and 3 is available from the corresponding author on reasonable request. The seismic reflection data analysed in Fig. 4 are available from Statoil ASA, but restrictions apply to the availability of these data, which were used under license for the current study, and so are not publicly available. Data are however available from the authors upon reasonable request and with permission of Statoil ASA.

## Electronic supplementary material

Supplementary Information
