# Supplementary material for: Virtual acoustics in inhomogeneous media with single-sided access
Source: Sci Rep. 2018 Feb 6;8:2497. doi: 10.1038/s41598-018-20924-x (PMC5802802; doi:10.1038/s41598-018-20924-x)
Supplement: Supplementary file 1 — Supplementary Information [file 41598_2018_20924_MOESM1_ESM.pdf]

Virtual acoustics in inhomogeneous media with single-sided access: Supplementary Information

Kees Wapenaar[1,*], Joeri Brackenhoff[1], Jan Thorbecke[1], Joost van der Neut[2], Evert Slob[1], and Eric Verschuur[2]

[1]Department of Geoscience and Engineering, Delft University of Technology, Stevinweg 1, 2628 CN Delft, The Netherlands
[2]Department of Imaging Physics, Delft University of Technology, Lorentzweg 1, 2628 CJ Delft, The Netherlands
[*]c.p.a.wapenaar@tudelft.nl

1 Classical representation of the homogeneous Green's function

1.1 Definition of the homogeneous Green's function

Consider an inhomogeneous lossless acoustic medium with compressibility $\kappa(\mathbf{x})$ and mass density $\rho(\mathbf{x})$. Here $\mathbf{x}$ denotes the Cartesian coordinate vector $\mathbf{x}=(x_1,x_2,x_3)$; the x_3 axis is pointing downward. In this medium a space $(\mathbf{x})$ and time (t) dependent source distribution $q(\mathbf{x},t)$ is present, with q defined as the volume-injection rate density. The acoustic wave field, caused by this source distribution, is described in terms of the acoustic pressure $p(\mathbf{x},t)$ and the particle velocity $v_i(\mathbf{x},t)$. These field quantities obey the equation of motion and the stress-strain relation, according to

$$\rho\partial_t v_i + \partial_i p = 0, \tag{1}$$

$$\kappa\partial_t p + \partial_i v_i = q. \tag{2}$$

Here ∂_t and ∂_i stand for the temporal and spatial differential operators $\partial/\partial t$ and $\partial/\partial x_i$, respectively. The summation convention applies to repeated subscripts. When q is an impulsive source at $\mathbf{x}=\mathbf{s}$ and $t=0$, according to

$$q(\mathbf{x},t)=\delta(\mathbf{x}-\mathbf{s})\delta(t), \tag{3}$$

then the causal solution of equations (1) and (2) defines the Green's function, hence

$$p(\mathbf{x},t)=G(\mathbf{x},\mathbf{s},t). \tag{4}$$

By eliminating v_i from equations (1) and (2) and substituting equations (3) and (4), we find that the Green's function $G(\mathbf{x},\mathbf{s},t)$ obeys the following wave equation

$$\partial_i(\rho^{-1}\partial_i G) - \kappa\partial_t^2 G = -\delta(\mathbf{x}-\mathbf{s})\partial_t\delta(t). \tag{5}$$

Wave equation (5) is symmetric in time, except for the source on the right-hand side, which is anti-symmetric. Hence, the time-reversed Green's function $G(\mathbf{x},\mathbf{s},-t)$ obeys the same wave equation, but with opposite sign for the source. By summing the wave equations for $G(\mathbf{x},\mathbf{s},t)$ and $G(\mathbf{x},\mathbf{s},-t)$, the sources on the right-hand sides cancel each other, hence, the function

$$G_{\rm h}(\mathbf{x},\mathbf{s},t)=G(\mathbf{x},\mathbf{s},t)+G(\mathbf{x},\mathbf{s},-t) \tag{6}$$

obeys the homogeneous equation

$$\partial_i(\rho^{-1}\partial_i G_{\rm h}) - \kappa\partial_t^2 G_{\rm h} = 0. \tag{7}$$

Therefore $G_{\rm h}(\mathbf{x},\mathbf{s},t)$, as defined in equation (6), is called the homogeneous Green's function.

1.2 Reciprocity theorems

We define the temporal Fourier transform of a space- and time-dependent quantity $p(\mathbf{x},t)$ as

$$p(\mathbf{x},\omega) = \int_{-\infty}^{\infty} p(\mathbf{x},t)\exp(-j\omega t)\mathrm{d}t, \tag{8}$$

where ω is the angular frequency and j the imaginary unit. To keep the notation simple, we denote quantities in the time and frequency domain by the same symbol. In the frequency domain, equations (1) and (2) transform to

$$j\omega\rho v_i + \partial_i p = 0, \tag{9}$$
$$j\omega\kappa p + \partial_i v_i = q. \tag{10}$$

We introduce two independent acoustic states, which will be distinguished by subscripts A and B. Rayleigh's reciprocity theorem is obtained by considering the quantity $\partial_i\{p_A v_{i,B} - v_{i,A} p_B\}$, applying the product rule for differentiation, substituting equations (9) and (10) for both states, integrating the result over a spatial domain $\mathbb{V}$ enclosed by boundary $\mathbb{S}$ with outward pointing normal n_i, and applying the theorem of Gauss.[1,2] Assuming that in $\mathbb{V}$ the medium parameters $\kappa(\mathbf{x})$ and $\rho(\mathbf{x})$ in the two states are identical, this yields Rayleigh's reciprocity theorem of the convolution type

$$\int_{\mathbb{V}}\{p_A q_B - q_A p_B\}\mathrm{d}\mathbf{x} = -\oint_{\mathbb{S}} \frac{1}{j\omega\rho}\{p_A\partial_i p_B - (\partial_i p_A)p_B\}n_i\mathrm{d}\mathbf{x}. \tag{11}$$

We derive a second form of Rayleigh's reciprocity theorem for time-reversed wave fields. In the frequency domain, time-reversal is replaced by complex conjugation. When p is a solution of equations (9) and (10) with source distribution q (and real-valued medium parameters), then p^* obeys the same equations with source distribution $-q^*$ (the superscript * denotes complex conjugation). Making these substitutions for state A in equation (11) we obtain Rayleigh's reciprocity theorem of the correlation type[3]

$$\int_{\mathbb{V}}\{p_A^* q_B + q_A^* p_B\}\mathrm{d}\mathbf{x} = -\oint_{\mathbb{S}} \frac{1}{j\omega\rho}\{p_A^*\partial_i p_B - (\partial_i p_A^*)p_B\}n_i\mathrm{d}\mathbf{x}. \tag{12}$$

1.3 Representation of the homogeneous Green's function

We choose point sources in both states, according to $q_A(\mathbf{x},\omega) = \delta(\mathbf{x}-\mathbf{s})$ and $q_B(\mathbf{x},\omega) = \delta(\mathbf{x}-\mathbf{r})$, with $\mathbf{s}$ and $\mathbf{r}$ both in $\mathbb{V}$. The fields in states A and B are thus expressed in terms of Green's functions, according to

$$p_A(\mathbf{x},\omega) = G(\mathbf{x},\mathbf{s},\omega), \tag{13}$$
$$p_B(\mathbf{x},\omega) = G(\mathbf{x},\mathbf{r},\omega), \tag{14}$$

with $G(\mathbf{x},\mathbf{s},\omega)$ and $G(\mathbf{x},\mathbf{r},\omega)$ being the Fourier transforms of $G(\mathbf{x},\mathbf{s},t)$ and $G(\mathbf{x},\mathbf{r},t)$, respectively. Making these substitutions in equation (12) and using source-receiver reciprocity of the Green's functions gives[4–7]

$$G_{\mathrm{h}}(\mathbf{r},\mathbf{s},\omega) = \oint_{\mathbb{S}} \frac{-1}{j\omega\rho(\mathbf{x})}\{\partial_i G(\mathbf{r},\mathbf{x},\omega)G^*(\mathbf{x},\mathbf{s},\omega) - G(\mathbf{r},\mathbf{x},\omega)\partial_i G^*(\mathbf{x},\mathbf{s},\omega)\}n_i\mathrm{d}\mathbf{x}, \tag{15}$$

where $G_{\mathrm{h}}(\mathbf{r},\mathbf{s},\omega)$ is the homogeneous Green's function in the frequency domain, defined as

$$G_{\mathrm{h}}(\mathbf{r},\mathbf{s},\omega) = G(\mathbf{r},\mathbf{s},\omega) + G^*(\mathbf{r},\mathbf{s},\omega) = 2\Re\{G(\mathbf{r},\mathbf{s},\omega)\}, \tag{16}$$

where $\Re$ denotes the real part. Equation (15) is an exact representation for the homogeneous Green's function $G_{\mathrm{h}}(\mathbf{r},\mathbf{s},\omega)$.

When $\mathbb{S}$ is sufficiently smooth and the medium outside $\mathbb{S}$ is homogeneous (with mass density ρ_0 and compressibility κ_0), the two terms under the integral in equation (15) are nearly identical (but with opposite signs), hence

$$G_{\mathrm{h}}(\mathbf{r},\mathbf{s},\omega) = \frac{2}{j\omega\rho_0}\oint_{\mathbb{S}} G(\mathbf{r},\mathbf{x},\omega)\partial_i G^*(\mathbf{x},\mathbf{s},\omega)n_i\mathrm{d}\mathbf{x}. \tag{17}$$

The main approximation is that evanescent waves are neglected at $\mathbb{S}$.[8] Using equations (9) and (13) this becomes

$$G_{\mathrm{h}}(\mathbf{r},\mathbf{s},\omega) = 2\oint_{\mathbb{S}} G(\mathbf{r},\mathbf{x},\omega)V^*(\mathbf{x},\mathbf{s},\omega)\mathrm{d}\mathbf{x}, \tag{18}$$

where $V(\mathbf{x},\mathbf{s},\omega) = v_i(\mathbf{x},\mathbf{s},\omega)n_i$ stands for the normal component of the particle velocity at $\mathbf{x}$ on $\mathbb{S}$, due to a source at $\mathbf{s}$. In the time domain this becomes

$$G(\mathbf{r},\mathbf{s},t) + G(\mathbf{r},\mathbf{s},-t) = 2\oint_{\mathbb{S}} G(\mathbf{r},\mathbf{x},t) * V(\mathbf{x},\mathbf{s},-t)\mathrm{d}\mathbf{x}, \tag{19}$$

where the inline asterisk ($*$) denotes temporal convolution. This is equation (1) in the main paper.

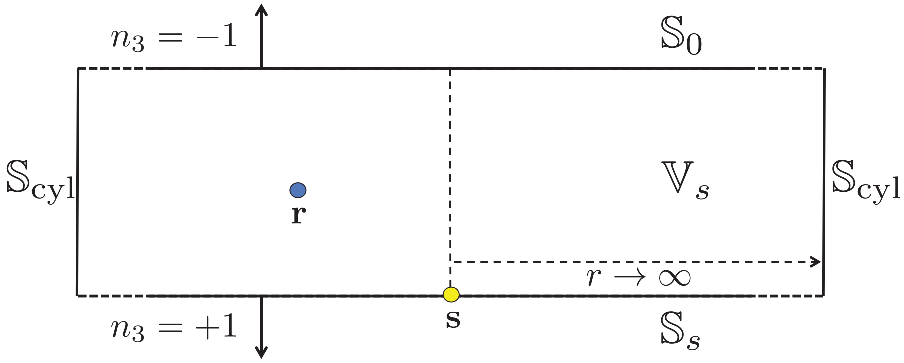

Figure S1. Modified domain $\mathbb{V}$, enclosed by $\mathbb{S}_0 \cup \mathbb{S}_s \cup \mathbb{S}_{\rm cyl}$ (side view).

2 Single-sided Green's function representations

2.1 Decomposed reciprocity theorems

For the derivation of the single-sided Green's function representations we define $\mathbb{V}_s$ as the domain enclosed by two horizontal boundaries $\mathbb{S}_0$ and $\mathbb{S}_s$, and a cylindrical boundary $\mathbb{S}_{\rm cyl}$ with infinite radius, see Figure S1. Here $\mathbb{S}_0$ is the accessible horizontal boundary of the medium where the measurements take place. It is defined by $x_3 = x_{3,0}$. We assume that the medium above $\mathbb{S}_0$ is homogeneous. Furthermore, $\mathbb{S}_s$ is a horizontal boundary at the depth of $\mathbf{s}$ and is defined by $x_3 = x_{3,s}$. The subscript s in $\mathbb{S}_s$ denotes that this boundary depends on the depth of $\mathbf{s}$. Consequently, $\mathbb{V}_s$ also depends on the depth of $\mathbf{s}$. Finally, $\mathbb{S}_{\rm cyl}$ is a cylindrical boundary with a vertical axis through $\mathbf{s}$ and infinite radius. This cylindrical boundary exists between $\mathbb{S}_0$ and $\mathbb{S}_s$ and closes the boundary $\mathbb{S}$.

The contribution of the boundary integral over $\mathbb{S}_{\rm cyl}$ in equations (11) and (12) vanishes.[9] This implies that we can restrict the integration to the boundaries $\mathbb{S}_0$ and $\mathbb{S}_s$. Note that $\mathbf{n} = (0,0,-1)$ on $\mathbb{S}_0$ and $\mathbf{n} = (0,0,+1)$ on $\mathbb{S}_s$. On the boundaries $\mathbb{S}_0$ and $\mathbb{S}_s$ we decompose the fields in both states into downgoing and upgoing fields, according to

$$p_A = p_A^+ + p_A^-, \tag{20}$$
$$p_B = p_B^+ + p_B^-, \tag{21}$$

where superscripts $+$ and $-$ stand for downgoing (i.e., propagating in the positive x_3-direction) and upgoing (i.e., propagating in the negative x_3-direction), respectively. Substituting these expressions into equations (11) and (12) and using the one-way wave equations for downgoing and upgoing waves at $\mathbb{S}_0$ and $\mathbb{S}_s$, we obtain[9]

$$\int_{\mathbb{V}_s} \{p_A q_B - q_A p_B\} \mathrm{d}\mathbf{x} = \int_{\mathbb{S}_0} \frac{-2}{j\omega\rho} \{(\partial_3 p_A^+) p_B^- + (\partial_3 p_A^-) p_B^+\} \mathrm{d}\mathbf{x} + \int_{\mathbb{S}_s} \frac{2}{j\omega\rho} \{(\partial_3 p_A^+) p_B^- + (\partial_3 p_A^-) p_B^+\} \mathrm{d}\mathbf{x}, \tag{22}$$

and

$$\int_{\mathbb{V}_s} \{p_A^* q_B + q_A^* p_B\} \mathrm{d}\mathbf{x} = \int_{\mathbb{S}_0} \frac{-2}{j\omega\rho} \{(\partial_3 p_A^+)^* p_B^+ + (\partial_3 p_A^-)^* p_B^-\} \mathrm{d}\mathbf{x} + \int_{\mathbb{S}_s} \frac{2}{j\omega\rho} \{(\partial_3 p_A^+)^* p_B^+ + (\partial_3 p_A^-)^* p_B^-\} \mathrm{d}\mathbf{x}, \tag{23}$$

respectively. In the latter equation, evanescent waves at $\mathbb{S}_0$ and $\mathbb{S}_s$ are ignored.

2.2 Single-sided representation of the homogeneous Green's function

We use reciprocity theorems (22) and (23) to derive a single-sided representation of the homogeneous Green's function $G_{\rm h}(\mathbf{r},\mathbf{s},\omega)$.

For state A we introduce a focusing function $f_1(\mathbf{x},\mathbf{s},\omega)$. Here $\mathbf{s}$ denotes the focal point; it lies at $\mathbb{S}_s$ which we will call the focal plane. The focusing function is defined in a source-free truncated medium, which is identical to the actual medium above the focal plane $\mathbb{S}_s$ but homogeneous below this plane, see Figure S2. For $\mathbf{x}$ on the boundaries $\mathbb{S}_0$ and $\mathbb{S}_s$ (and in the homogeneous half-spaces above $\mathbb{S}_0$ and below $\mathbb{S}_s$), the focusing function is written as a superposition of downgoing and upgoing components, according to

$$f_1(\mathbf{x},\mathbf{s},\omega) = f_1^+(\mathbf{x},\mathbf{s},\omega) + f_1^-(\mathbf{x},\mathbf{s},\omega). \tag{24}$$

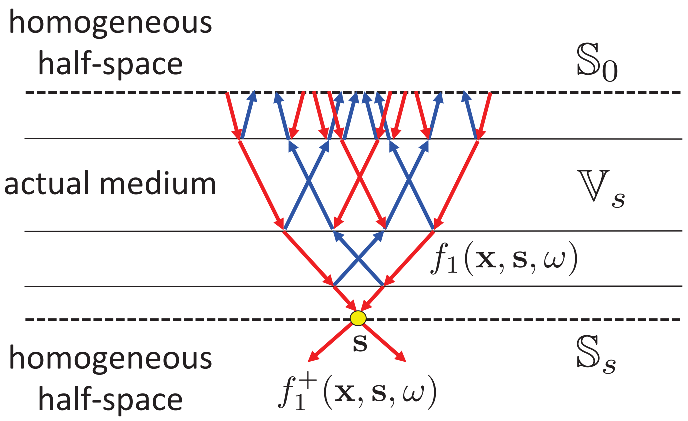

Figure S2. Illustration of the focusing function $f_1(\mathbf{x},\mathbf{s},\omega)$, defined in a truncated version of the actual medium.

The downgoing focusing function f_1^+ is incident to the inhomogeneous medium from the homogeneous half-space above $\mathbb{S}_0$, and the upgoing function f_1^- is its response. The focusing function propagates and scatters in the truncated medium in $\mathbb{V}_s$, focuses at $\mathbf{s}$ on $\mathbb{S}_s$, and continues as a downgoing wave field f_1^+ in the homogeneous lossless half-space below $\mathbb{S}_s$. The focusing conditions at the focal plane $\mathbb{S}_s$ are defined as

$$[\partial_3 f_1^+(\mathbf{x},\mathbf{s},\omega)]_{x_3=x_{3,s}} = -\tfrac{1}{2}j\omega\rho(\mathbf{s})\delta(\mathbf{x}_\mathrm{H}-\mathbf{s}_\mathrm{H}), \tag{25}$$

$$[\partial_3 f_1^-(\mathbf{x},\mathbf{s},\omega)]_{x_3=x_{3,s}} = 0. \tag{26}$$

Here $\mathbf{x}_\mathrm{H}$ and $\mathbf{s}_\mathrm{H}$ stand for the horizontal coordinates of $\mathbf{x}$ and $\mathbf{s}$, respectively.

For state B we take again the Green's function $G(\mathbf{x},\mathbf{r},\omega)$, which, for $\mathbf{x}$ at $\mathbb{S}_0$ and $\mathbb{S}_s$, is written as

$$G(\mathbf{x},\mathbf{r},\omega) = G^+(\mathbf{x},\mathbf{r},\omega) + G^-(\mathbf{x},\mathbf{r},\omega). \tag{27}$$

Here $\mathbf{r}$ can be chosen anywhere below the upper boundary $\mathbb{S}_0$. When $\mathbf{r}$ lies above $\mathbf{s}$ (and below $\mathbb{S}_0$, as illustrated in Figure S1), it is by definition situated in $\mathbb{V}_s$. When $\mathbf{r}$ lies below $\mathbf{s}$, it is situated outside $\mathbb{V}_s$. Because the upper half-space above $\mathbb{S}_0$ is homogeneous, we have

$$[G^+(\mathbf{x},\mathbf{r},\omega)]_{x_3=x_{3,0}} = 0. \tag{28}$$

Substituting $p_A(\mathbf{x},\omega) = f_1(\mathbf{x},\mathbf{s},\omega)$, $p_A^\pm(\mathbf{x},\omega) = f_1^\pm(\mathbf{x},\mathbf{s},\omega)$, $q_A(\mathbf{x},\omega) = 0$, $p_B^\pm(\mathbf{x},\omega) = G^\pm(\mathbf{x},\mathbf{r},\omega)$ and $q_B(\mathbf{x},\omega) = \delta(\mathbf{x}-\mathbf{r})$ into equations (22) and (23), using equations (25), (26) and (28), gives

$$G^-(\mathbf{s},\mathbf{r},\omega) + \chi_s(\mathbf{r})f_1(\mathbf{r},\mathbf{s},\omega) = -\int_{\mathbb{S}_0} \frac{2}{j\omega\rho(\mathbf{x})}\{\partial_3 f_1^+(\mathbf{x},\mathbf{s},\omega)\}G^-(\mathbf{x},\mathbf{r},\omega)\mathrm{d}\mathbf{x} \tag{29}$$

and

$$G^+(\mathbf{s},\mathbf{r},\omega) - \chi_s(\mathbf{r})f_1^*(\mathbf{r},\mathbf{s},\omega) = \int_{\mathbb{S}_0} \frac{2}{j\omega\rho(\mathbf{x})}\{\partial_3 f_1^-(\mathbf{x},\mathbf{s},\omega)\}^* G^-(\mathbf{x},\mathbf{r},\omega)\mathrm{d}\mathbf{x}, \tag{30}$$

where χ_s is the characteristic function of the domain $\mathbb{V}_s$. It is defined as

$$\chi_s(\mathbf{r}) = \begin{cases} 1, & \text{for } \mathbf{r} \text{ in } \mathbb{V}_s, \\ \frac{1}{2}, & \text{for } \mathbf{r} \text{ on } \mathbb{S} = \mathbb{S}_0 \cup \mathbb{S}_s, \\ 0, & \text{for } \mathbf{r} \text{ outside } \mathbb{V}_s \cup \mathbb{S}. \end{cases} \tag{31}$$

Summing equations (29) and (30), using $G(\mathbf{x},\mathbf{r},\omega) = G^-(\mathbf{x},\mathbf{r},\omega)$ for $\mathbf{x}$ at $\mathbb{S}_0$, and using source-receiver reciprocity for the Green's functions, yields

$$G(\mathbf{r},\mathbf{s},\omega) + \chi_s(\mathbf{r})2j\Im\{f_1(\mathbf{r},\mathbf{s},\omega)\} = \int_{\mathbb{S}_0} G(\mathbf{r},\mathbf{x},\omega)F(\mathbf{x},\mathbf{s},\omega)\mathrm{d}\mathbf{x}, \tag{32}$$

with

$$F(\mathbf{x},\mathbf{s},\omega) = -\frac{2}{j\omega\rho(\mathbf{x})}\partial_3\big(f_1^+(\mathbf{x},\mathbf{s},\omega) - \{f_1^-(\mathbf{x},\mathbf{s},\omega)\}^*\big), \tag{33}$$

where $\Im$ denotes the imaginary part. Inverse Fourier transforming equation (32) gives

$$G(\mathbf{r},\mathbf{s},t) + \chi_s(\mathbf{r})\{f_1(\mathbf{r},\mathbf{s},t) - f_1(\mathbf{r},\mathbf{s},-t)\} = \int_{\mathbb{S}_0} G(\mathbf{r},\mathbf{x},t) * F(\mathbf{x},\mathbf{s},t)\mathrm{d}\mathbf{x}. \tag{34}$$

This is equation (2) in the main paper. Taking two times the real part of both sides of equation (32) gives

$$G_\mathrm{h}(\mathbf{r},\mathbf{s},\omega) = 2\Re\int_{\mathbb{S}_0} G(\mathbf{r},\mathbf{x},\omega)F(\mathbf{x},\mathbf{s},\omega)\mathrm{d}\mathbf{x}. \tag{35}$$

In the time domain this becomes

$$G(\mathbf{r},\mathbf{s},t) + G(\mathbf{r},\mathbf{s},-t) = \int_{\mathbb{S}_0} G(\mathbf{r},\mathbf{x},t) * F(\mathbf{x},\mathbf{s},t)\mathrm{d}\mathbf{x} + \int_{\mathbb{S}_0} G(\mathbf{r},\mathbf{x},-t) * F(\mathbf{x},\mathbf{s},-t)\mathrm{d}\mathbf{x}. \tag{36}$$

This is equation (3) in the main paper.

Note that the Green's function $G(\mathbf{r},\mathbf{x},\omega)$ on the right-hand side of equation (35) can be obtained from a similar representation. To see this, replace in the right-hand side of equation (35) $\mathbb{S}_0$ by $\mathbb{S}_0'$ just above $\mathbb{S}_0$, replace $\mathbf{x}$ on $\mathbb{S}_0$ by $\mathbf{x}'$ on $\mathbb{S}_0'$, $\mathbf{r}$ inside the medium by $\mathbf{x}$ on $\mathbb{S}_0$ and $\mathbf{s}$ by $\mathbf{r}$. This gives a representation for $G_\mathrm{h}(\mathbf{x},\mathbf{r},\omega)$. Using source-receiver reciprocity we finally get

$$G_\mathrm{h}(\mathbf{r},\mathbf{x},\omega) = 2\Re\int_{\mathbb{S}_0'} G(\mathbf{x}',\mathbf{x},\omega)F(\mathbf{x}',\mathbf{r},\omega)\mathrm{d}\mathbf{x}'. \tag{37}$$

In the time domain this becomes

$$G(\mathbf{r},\mathbf{x},t) + G(\mathbf{r},\mathbf{x},-t) = \int_{\mathbb{S}_0'} G(\mathbf{x}',\mathbf{x},t) * F(\mathbf{x}',\mathbf{r},t)\mathrm{d}\mathbf{x}' + \int_{\mathbb{S}_0'} G(\mathbf{x}',\mathbf{x},-t) * F(\mathbf{x}',\mathbf{r},-t)\mathrm{d}\mathbf{x}'. \tag{38}$$

This is equation (4) in the main paper, where for simplicity the prime in $\mathbb{S}_0'$ is dropped.

References

1. de Hoop, A. T. Time-domain reciprocity theorems for acoustic wave fields in fluids with relaxation. *J. Acoust. Soc. Am.* **84**, 1877–1882 (1988).

2. Fokkema, J. T. & van den Berg, P. M. *Seismic applications of acoustic reciprocity* (Elsevier, Amsterdam, 1993).

3. Bojarski, N. N. Generalized reaction principles and reciprocity theorems for the wave equations, and the relationship between the time-advanced and time-retarded fields. *J. Acoust. Soc. Am.* **74**, 281–285 (1983).

4. Porter, R. P. Diffraction-limited, scalar image formation with holograms of arbitrary shape. *J. Opt. Soc. Am.* **60**, 1051–1059 (1970).

5. Oristaglio, M. L. An inverse scattering formula that uses all the data. *Inverse Probl.* **5**, 1097–1105 (1989).

6. Wapenaar, K. Retrieving the elastodynamic Green's function of an arbitrary inhomogeneous medium by cross correlation. *Phys. Rev. Lett.* **93**, 254301 (2004).

7. van Manen, D.-J., Robertsson, J. O. A. & Curtis, A. Modeling of wave propagation in inhomogeneous media. *Phys. Rev. Lett.* **94**, 164301 (2005).

8. Wapenaar, K. *et al.* Seismic interferometry by crosscorrelation and by multidimensional deconvolution: a systematic comparison. *Geophys. J. Int.* **185**, 1335–1364 (2011).

9. Wapenaar, C. P. A. & Berkhout, A. J. *Elastic wave field extrapolation* (Elsevier, Amsterdam, 1989).
